# *Zingiber officinale* Root Capsule Extract Synergistically Enhance the Anti-Inflammatory Effects of Diclofenac Sodium in Experimental Acute Inflammation

**DOI:** 10.3390/ijms25031781

**Published:** 2024-02-01

**Authors:** Ioana Boarescu, Paul-Mihai Boarescu, Raluca Maria Pop, Ioana Corina Bocșan, Dan Gheban, Adriana Elena Bulboacă, Anca Dana Buzoianu, Sorana D. Bolboacă

**Affiliations:** 1 Department of Medical Informatics and Biostatistics, Iuliu Haţieganu University of Medicine and Pharmacy Cluj-Napoca, Louis Pasteur Street, No. 6, 400349 Cluj-Napoca, Romania; 2 Department of Biomedical Sciences, Faculty of Medicine and Biological Sciences, “Ștefan cel Mare” University of Suceava, 720229 Suceava, Romania; 3 Department of Pharmacology, Toxicology and Clinical Pharmacology, Iuliu Haţieganu University of Medicine and Pharmacy Cluj-Napoca, Gheorghe Marinescu Street, No. 23, 400337 Cluj-Napoca, Romania; 4 Department of Pathological Anatomy, Iuliu Haţieganu University of Medicine and Pharmacy Cluj-Napoca, Clinicilor Street, No. 3–5, 400006 Cluj-Napoca, Romania; 5 Department of Pathophysiology, Iuliu Haţieganu University of Medicine and Pharmacy Cluj-Napoca, Victor Babeş Street, No. 2–4, 400012 Cluj-Napoca, Romania

**Keywords:** diclofenac sodium, carrageenan, inflammation, cytokines, ginger root, extract

## Abstract

The present study aimed to evaluate the anti-inflammatory effects of ginger (*Zingiber officinale*) root capsule extract (GRCE) in doses of 100 mg/kg b.w. (body weight) and 200 mg/kg b.w. alone and in combination with a low dose (5 mg/kg b.w.) of diclofenac sodium (D) on carrageenan-induced acute inflammation (AI). The association of GRCE in a dose of 200 mg/kg b.w. with D offered the highest inhibition percentage for edema, reaching the maximum level of inhibition (95%) after 24 h. The association of GRCE in a dose of 200 mg/kg b.w. with D showed the ability to reduce tissue inflammatory changes when compared to D alone, while GRCE alone did not exhibit such properties. The association of both doses of GRCE with D showed significantly lower plasma and tissue levels of pro-inflammatory cytokines such as tumor necrosis factor-α (TNF-α), interleukin-6 (IL-6), and interleukin-1β (IL-1β) by up to 55% (*p* ≤ 0.0317), with the best results obtained by the group who received GRCE in the higher dose. These associations reduced the serum and tissue levels of prostaglandin-endoperoxide synthase 2 (COX-2) by up to 71% (*p* ≤ 0.0371). In conclusion, the association of GRCE with a low dose of D could be an appropriate combination to decrease the dose used to reduce serum and tissue levels of inflammatory molecules, edema, and histological changes in acute inflammation. Further research will be necessary to achieve clinical evaluation.

## 1. Introduction

Inflammation is a broad and ancient medical term referring to a set of classic signs and symptoms, including pain, edema, erythema, warmness, and loss of function [1]. It is characterized by a set of complex changing responses to tissue injury primarily caused by harmful stimuli, such as pathogens, damaged cells, toxic compounds, or irradiation [2].

Carrageenan is a high-molecular-weight sulphated proinflammatory polysaccharide that is often used to induce local inflammation (paw edema) [2]. Carrageenan-induced hind paw edema is a common animal model used to evaluate the anti-inflammatory effects of different drugs or active compounds [3,4]. The early phase of inflammation is observed at around 1 h after the administration of carrageenan and is related to the release of bradykinin, serotonin, histamine, and, to a lesser extent, prostaglandins produced by cyclooxygenase (COX) enzymes. The delayed phase (after 1 h) is attributed to the continuation of prostaglandin generation and neutrophil infiltration [5], and the release of pro-inflammatory cytokines such as tumor necrosis factor (TNF-α) or interleukin-1 β (IL-1 β). The overproduction of the neutrophil-derived free radicals and nitric oxide (NO) are also involved in the delayed phase of carrageenan-induced acute inflammation [6]. It was suggested that drugs targeting COX enzymes, pro-inflammatory protein expression (e.g., inducible nitric oxide synthase: iNOS), and free radical formation might provide better control of the inflammation process [7].

Non-Steroidal Anti-Inflammatory Drugs (NSAIDs) are one of the most commonly prescribed classes of medication for acute and chronic inflammation. Their major therapeutic actions are related to their ability to block the synthesis of certain prostaglandins (PGs) through the inhibition of cyclooxygenase enzymes (COX-1 and COX-2) [8]. COX-1 enzyme is expressed in normal cells and produces thromboxane A2 and PGs, which control renal homeostasis, platelet aggregation, the mucosal barrier in the gastrointestinal tract, and possess other physiological functions. COX-2 is induced in inflammatory cells and produces PGs that are related to inflammation, pain, and fever. The inhibition of COX-2 most likely represents the desired effect of NSAIDs by providing anti-inflammatory, analgesic and antipyretic responses, while the inhibition of the COX-1 enzyme plays a major role in undesired side effects such as renal toxicities or injury to the gastro-intestinal mucosa [9]. Therefore, the administration of NSAIDs may cause acute renal failure, gastrointestinal ulcers, hypertension, the worsening of preexisting heart failure, and even serious cardiovascular events. These adverse effects may be prevented by limiting the NSAID dosage and duration of administration and also by performing risk assessments for each patient depending on the associated pathology [10].

Diclofenac is a nonselective NSAID widely used as an anti-inflammatory, antipyretic, and analgesic drug, but it has a reduced bioavailability due to hepatic first-pass metabolism and is associated with many adverse effects on the gastrointestinal, hepatic, renal, and cardiovascular systems [11,12].

Ginger (*Zingiber officinale*) has been commonly used as a spice and as a herbal medicine for a long time; ginger root is used to attenuate and treat several common diseases, such as colds, nausea, headaches, and emesis [13]. It has been observed to be rich in various chemical constituents, including phenolic compounds, polysaccharides, terpenes, lipids, organic acids, and raw fiber [13]. The health benefits of ginger are mainly attributed to its phenolic compounds, such as gingerols and shogaols [14]. Recent research has demonstrated the anti-inflammatory [15], antimicrobial [16], antioxidant [17], and anticancer [18] effects of ginger.

Ginger was observed to possess inhibitory effects on prostaglandin biosynthesis, suggesting that this natural product shares pharmacological properties with non-steroidal anti-inflammatory drugs (NSAIDs) [19]. Moreover, gingerols were observed to alleviate inflammation by decreasing the number of proinflammatory cytokines and by increasing the number of anti-inflammatory cytokines, as a result of their ability to inhibit the activation of protein kinase B (Akt) and nuclear factor kappa B (NF-κB) signaling pathways [20].

We previously observed that ginger (*Zingiber officinale*) root capsule extract (GRCE) potentiates the analgesic and antioxidant effects of diclofenac sodium in carrageenan-induced paw edema [21]. In the present study, we aimed to evaluate the anti-inflammatory effect of ginger (*Zingiber officinale*) root capsule extract when associated with diclofenac sodium in carrageenan-induced acute inflammation, as we assumed that GRCE might offer additional anti-inflammatory effects when compared to NSAIDs.

## 2. Results

We performed statistical analysis on all of the rats included in this study since we did not encounter any incidents during the experiment.

### 2.1. Results on Serum Biomarkers

**Figure 1:**
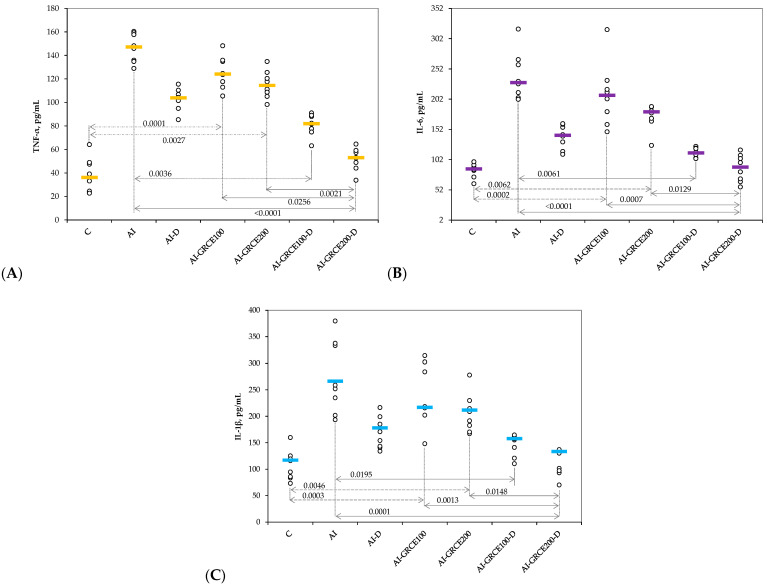
Variation in serum levels of (**A**) Tumor necrosis factor α (TNF-α), (**B**) Interleukin 6 (IL-6), and (**C**) Interleukin 1β (IL-1β) by groups. Notes: The circles represent the individual values, and the horizontal line is given by the median. Abbreviations: C—Control; AI—Acute inflammation; D—Diclofenac sodium; GRCE—Ginger root capsule extract.

**Figure 2:**
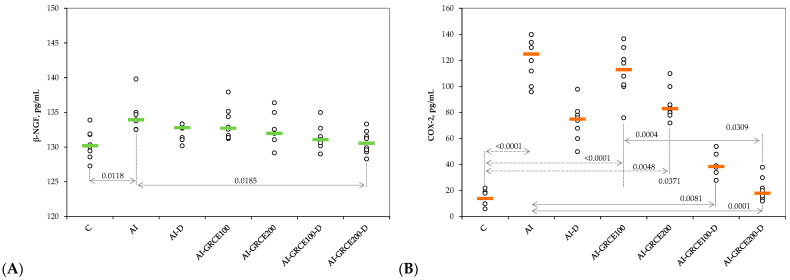
Variation in serum levels of (**A**) Serum values of β-Nerve Growth Factor (β-NGF) and (**B**) Prostaglandin-endoperoxide synthase 2 (COX-2) by groups. Notes: The circles represent the individual values, and the horizontal line is given by the median. Abbreviations: C—Control; AI—Acute inflammation; D—Diclofenac sodium; GRCE—Ginger root capsule extract.

The serum levels of pro-inflammatory cytokines and prostaglandin-endoperoxide synthase 2 (COX-2) increased after AI induction and the closest value to the control group was obtained when the highest dose of GRCE was associated with D (Figure 1 and Figure 2, Supplementary Material Tables S1 and S2).

### 2.2. Results on Tissue Biomarkers

**Figure 3:**
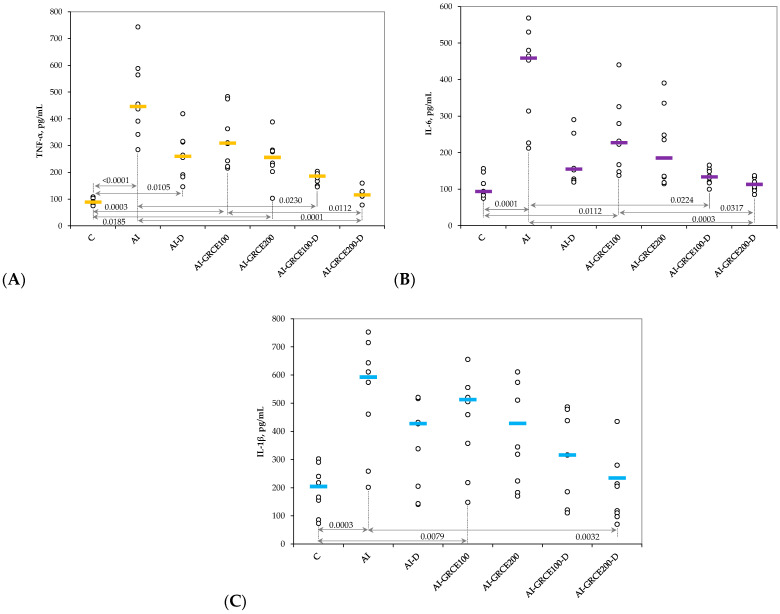
Variation in tissue levels of (**A**) Tumor necrosis factor α (TNF-α), (**B**) Interleukin 6 (IL-6), and (**C**) Interleukin 1β (IL-1β) by groups. Notes: The circles represent the individual values, and the horizontal line is given by the median. Abbreviations: C—Control; AI—Acute inflammation; D—Diclofenac sodium; GRCE—Ginger root capsule extract.

**Figure 4:**
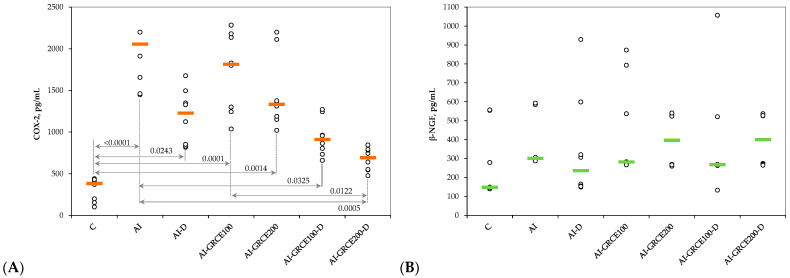
Variation in tissue levels of (**A**) Prostaglandin-endoperoxide synthase 2 (COX-2) and (**B**) β-Nerve Growth Factor (β-NGF) by groups. Notes: The circles represent the individual values, and the horizontal line is given by the median. Abbreviations: C—Control; AI—Acute inflammation; D—Diclofenac sodium; GRCE—Ginger root capsule extract.

Similar to serum levels, the tissue levels of pro-inflammatory cytokines and prostaglandin-endoperoxide synthase 2 (COX-2) increased after AI induction and the closest value to the control group was obtained when the highest dose of GRCE was associated with D (Figure 3 and Figure 4, Supplementary Material Tables S3 and S4).

### 2.3. Edema Reduction

**Figure 5:**
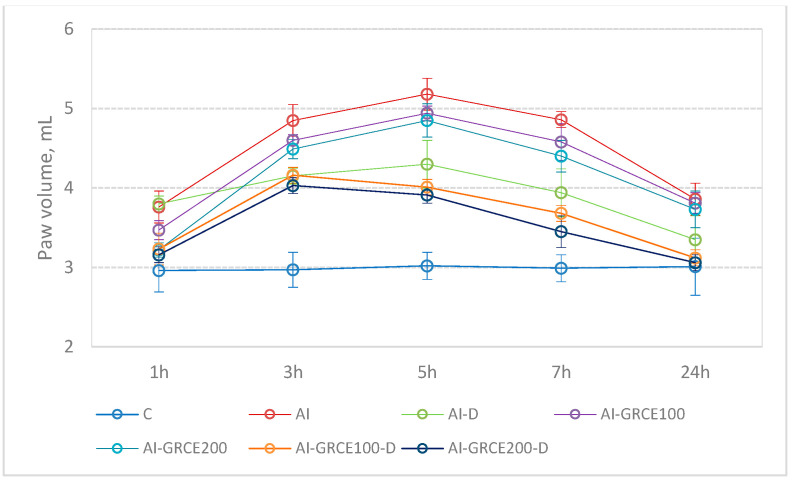

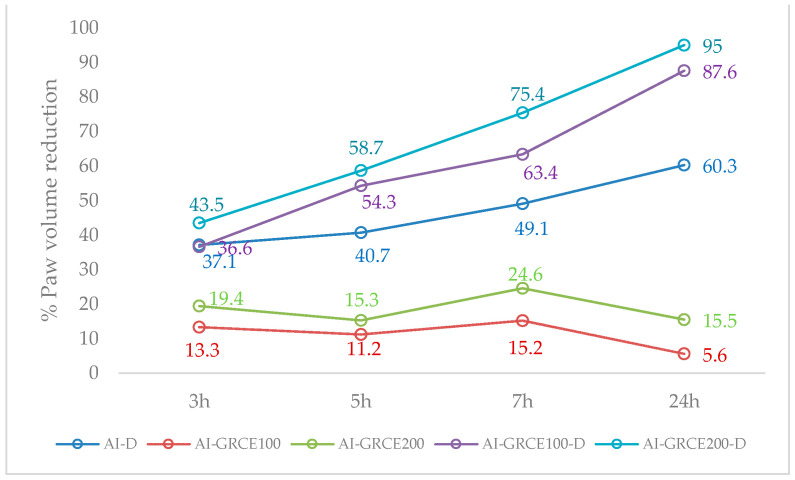
(**top**) Distribution of paw volume by group; (**bottom**) Distribution of percentage reduction of paw volume by group. The circle represents the arithmetic mean, and the whiskers are given by the value of standard deviation. Abbreviations: C—Control; AI—Acute inflammation; D—Diclofenac sodium; GRCE—Ginger root capsule extract.

Edema reduction is dose dependent and the best effect was obtained when GRCE was added to D (Figure 5).

Similar paw volumes were observed for AI (*p*-value > 0.9999) for the AI-GRCE100-D (*p*-value > 0.9999) and for the AI-GRCE200-D (*p*-value > 0.9999) groups, with values at 24 h not statistically significant compared to the C group. Compared to the AI group, the paw volume at 24 h proved statistically significantly lower in the AI-GRCE100-D group (*p*-value = 0.0072) and the AI-GRCE200-D group (*p*-value = 0.0072). At the end of the experiment, the paw volumes were lower in the AI-GRCE200-D group compared to the AI-GRCE100 group (*p*-value = 0.0049) and the AI-GRCE200 group (*p*-value = 0.0166).

### 2.4. Histological Assessment

**Figure 6:**
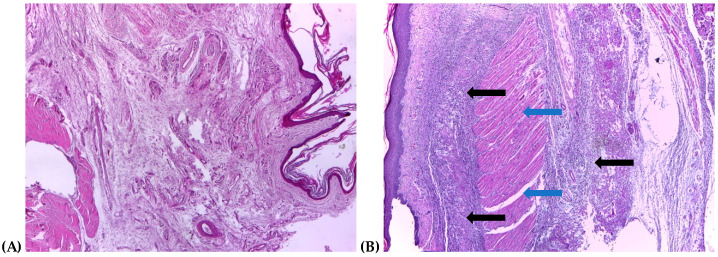

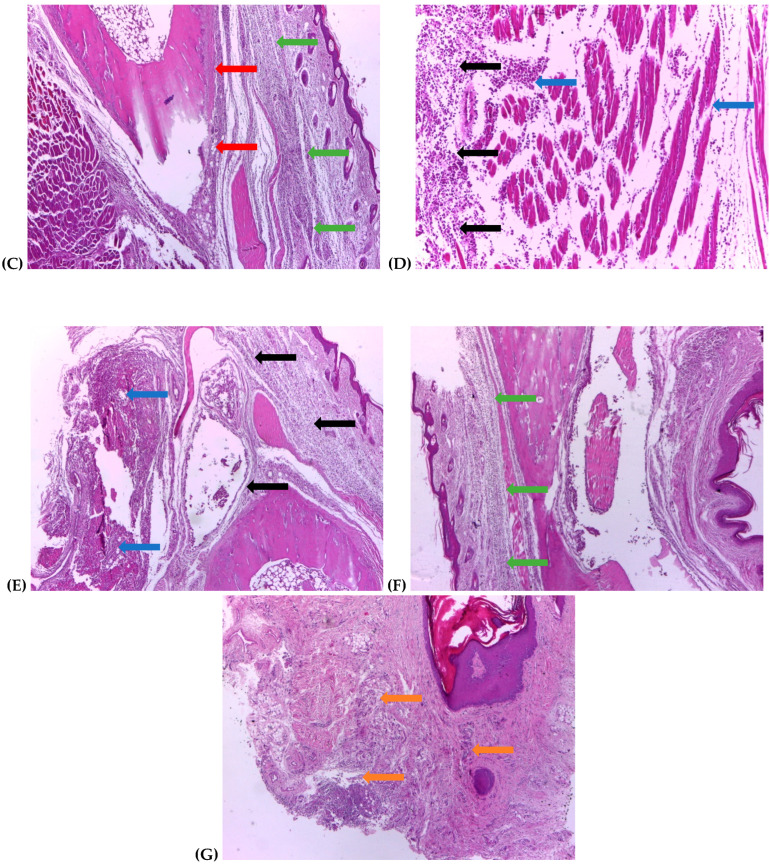
Histopathological examinations of the right hind paw (Hematoxylin & Eosin, ×100): (**A**) C: normal architecture of dermis and hypodermis of the right hind paw; (**B**) AI: severe acute purulent inflammation in the dermis and hypodermis (black arrow), with an extension on the underlying muscular tissue (blue arrow); (**C**) AID: moderate acute purulent inflammation in the dermis and hypodermis (green arrow), with fasciitis (red arrow); (**D**) AI-GRCE100: severe acute purulent inflammation in the dermis and hypodermis (black arrow), with an extension on the underlying muscular tissue (blue arrow); (**E**) AI-GRCE200: severe acute purulent inflammation in the dermis and hypodermis (black arrow), with an extension on the underlying muscular tissue (blue arrow); (**F**) AI-GRCE100-D: moderate acute purulent inflammation in the dermis and hypodermis (green arrow); (**G**) AI-GRCE100-D: reduced level of inflammation on dermis and hypodermis (orange arrow).

The histopathological examination of the hind paw showed the normal structure of the dermis and hypodermis in the control group (Figure 6A). Severe inflammation was observed in the AI group, which was characterized by acute purulent inflammation in the dermis and hypodermis that extended to the underlying muscular tissue (Figure 6B). In the D group, the histological examination revealed moderate acute purulent inflammation in the dermis and hypodermis, with associated fasciitis (Figure 6C). Both the AI-GRCE100 (Figure 6D) and the AI-GRCE200 (Figure 6E) groups exhibited comparable histopathological changes, suggesting that the administration of GRCE alone had no impact on the histopathological aspects of the inflamed hind paw. A lower GRCE dose associated with diclofenac sodium (AI-GRCE100-D group) led to moderate inflammation on the dermis and hypodermis (Figure 6F), while a higher dose led to a reduced level of inflammation on the dermis and hypodermis (Figure 6G).

## 3. Discussion

### 3.1. Serum and Tissue Biomarkers

The results of our study demonstrated that the association of ginger root capsule extract with diclofenac sodium could potentiate the anti-inflammatory effects of diclofenac sodium in reduced doses, with the best results obtained for GRCE in a dose of 200 mg/kg b.w. (Figure 1, Figure 2, Figure 3 and Figure 4, Supplementary Material Tables S1–S4).

TNF-α has been intrinsically linked with both phases of the edematogenic response in acute inflammation [22]. It is an inflammatory cytokine in acute inflammation, which is produced by macrophages/monocytes [23,24]. By expressing adhesion molecules, TNF-α participates in leukocyte adhesion to the epithelium, vasodilatation, and edema formation [25]. This pro-inflammatory cytokine has also been shown to promote collateral cytotoxicity in carrageenan-induced mouse paw edema by acting as an inducer of NO formation, a stimulator of prostaglandin, and an activator of the NF-κB signal transduction pathway [22].

Interleukin-6 is a pro-inflammatory cytokine produced by different cells that are expressed during states of cellular stress, such as cancer, infection or inflammation [26]. During the inflammation process, IL-6 is produced in the first phase at the site of inflammation and afterwards it moves to the liver through the bloodstream. When it reaches the liver, it leads to the rapid synthesis of an extensive range of acute phase proteins such as fibrinogen, haptoglobin, C-reactive protein (CRP), serum amyloid A, and α1-antichymotrypsin [27]. In carrageenan-induced paw edema, increased levels of IL-6 at the site of carrageenan administration facilitate the leukocyte recruitment and mediate edema formation [28].

Interleukin-1 α and β are other prototypic pro-inflammatory cytokines that play key roles in acute and chronic inflammatory, autoimmune disorders [29,30]. IL-1β together with TNF-α is responsible for several local and systemic characteristics of the inflammatory reaction, such as neutrophil accumulation at the injury site, the induction of vascular adhesion molecules, the stimulation of acute phase protein synthesis and increased body temperature [31]. Moreover, IL-1β also positively regulates the expression of cyclooxygenase 2 (COX-2) and therefore is involved in the production of prostaglandins, which are relevant in the inflammatory response [32].

Diclofenac sodium, like other NSAIDs, may inhibit the COX enzymes leading to a reduction in the levels of pro-inflammatory cytokines. As a result of COX-1 and COX-2 inhibition, D may also reduce the production of thromboxanes and prostaglandins, which play important roles in the inflammatory response [33]. Moreover, it has already been demonstrated that NSAIDs can reduce the production of cytokines by inhibiting the levels of TNF-α and IL-6, by interacting with transcriptional factors [34].

Ginger active compounds such as 6-gingerol and 6-shogaol were observed to have anti-inflammatory effects by inhibiting the production of inflammatory mediators, such as prostaglandin E2, inflammatory cytokines (TNF-α), interleukin-1β (IL-1β), and pro-inflammatory transcription factor (NF-κB). They also provide antioxidant effects as a result of a reduction in NO production and substantial free-radical scavenging activities. [35]. Ginger was observed to significantly inhibit the production of IFN-γ and IL-6 and suppress NF-κB via the degradation of the nuclear factor of the kappa light polypeptide gene enhancer in B-cells inhibitor, and alpha (IκB-α) in a lipopolysaccharide-induced inflammation in a mouse model [36].

Ezzat et al. analyzed the inflammatory mediators in edema exudates induced by carrageenan administration and they observed that the oral treatment with the crude hydralcoholic extract of ginger rhizomes resulted in significantly reduced levels of PGE2, TNF, Il6, IL-1β, and interferon γ, the macrophage inflammatory protein (MIP), or chemokine monocyte chemoattractant protein 1 (MCP-1), and regulated on activation, normal T cell expressed and secreted chemokine (RANTES) [37].

Gad [38] demonstrated that a dose of 400 mg of aqueous ginger extract can serve as an anti-inflammatory agent, diminishing the serum levels of NO, TNF-α, and IL-4 in albino rats with carrageenan-induced paw edema.

Nerve growth factor (NGF) is a member of the neurotrophic factor family, which is widely distributed in central nervous cells, peripheral Schwann cells, skeletal muscles, and glands. It can influence the differentiation, growth, development and survival of peripheral and central nerves and plays an important role in regulating the expression of its functional properties [39]. NGF also plays an important role in immune response and inflammation, as it is regarded as a painful inflammatory mediator [40]. Nerve Growth Factor Beta Subunit (β-NGF) contributes to the release of pain mediators such as histamine and prostaglandin from mast cells and is a crucial component for the sensitization of primary afferent nociceptors associated with tissue inflammation. A previous study reported that the increase in β-NGF upon carrageenan administration may facilitate the transmission of pain signals associated with inflammation [28].

Another study on carrageenan-induced AI performed by Amann and Schuligoi reported that NGF was increased in the inflamed paw, while the values detected in the contralateral paw skin were within the range found in normal rats. They observed that a low dose of indomethacin, another NSAID, reduces inflammatory edema but does not necessarily have consequences on increased NGF levels in inflamed hind paws [41].

Prostaglandin-endoperoxide synthase 2, also known as COX2, is an enzyme that is responsible for the production of prostaglandins during inflammation. NSAIDs are COX inhibitors that prevent the conversion of arachidonic acid to the inflammatory prostaglandins [42]. Diclofenac sodium was observed to possess dose-response relationships for COX-2 and COX-1 inhibition, with greater COX-2 selectivity [43]

The gingerols from ZO were demonstrated to exhibit a potent inhibition of COX-2 in a structure and concentration-dependent manner. Moreover, the findings suggest that gingerol and its synthetic analogues might have potential effects on other COX-2 inhibitors, suggesting that ginger could be useful to treat inflammation and pain [44].

To the best of our knowledge, this is the first study that has evaluated the effects of GRCE in addition to diclofenac sodium on the serum and tissue levels of TNF-α, IL-1β, IL-, β-NGF, and COX-2 in carrageenan-induced paw edema.

### 3.2. Edema Reduction

The results of the present study proved that the administration of diclofenac sodium led to edema reduction after the first hour. The association of GRCE with diclofenac sodium reduced paw edema earlier than with diclofenac sodium alone (Figure 5).

Diclofenac sodium significantly reduced paw edema due to the inhibition of COX-2 and the reduction in prostaglandins synthesis, which are pharmacological effects characteristic of nonsteroidal anti-inflammatory drugs (NSAIDs) [45]. The observed antiedematos effect of diclofenac sodium only after the first hour might be explained by the fact that the release of prostaglandins is characteristic of the second phase of edema [46].

Extracts of *Zingiber officinale* were already observed to reduce rat paw edema induced by carrageenan, similar to our results. Penna et al. observed that the crude hydralcoholic extract of ginger rhizomes reduced rat paw and skin edema induced by carrageenan. The mechanism of this inhibition remains unclear, but they showed that serotonin receptor antagonism could be an explanation for this effect [47]. The paw volume was better reduced in rats treated with GRCE in the first hour compared to diclofenac sodium, as the first phase of carrageenan-induced edema is characterized by the release of histamine, serotonin, and bradykinin [48]. Ezzat et al. [37], with an aqueous ginger extract used in a carrageenan-induced rat paw edema model, revealed that oral application significantly reduced the paw volume in a dose-dependent manner.

### 3.3. Histological Changes

In the present study, no histopathological differences were found between the AI group and the groups treated with GRCE in both doses (Figure 6). In contrast, the study performed by Zammel et al. concluded that the treatment for one week before carrageenan-induced inflammation alleviated the histopathological features in a dose of 100 mg/kg b.w. [49]. Diclofenac sodium slightly reduced the inflammatory process, similar to the results reported by Abdelhameed et al. [50]. Even with a higher dose of diclofenac sodium (30 mg/kg b.w.), they reported that the oral or topical administration of diclofenac sodium did not have significant effects on the histological changes in carrageenan-induced paw edema [50]. The association of GRCE with diclofenac sodium better reduced the inflammation level, with the best results obtained for the dose of 200 mg/kg b.w. (Figure 6). This is most probably because ginger can enhance the anti-inflammatory effect of diclofenac sodium by providing a supplementary reduction in the pro-inflammatory molecules.

## 4. Materials and Methods

### 4.1. Chemicals and Drugs

Ginger root capsule extract (GRCE) (Solaray, Park City, UT, USA) was purchased from a local pharmacy, as a food supplement sold in 250 mg capsules. As stated in the pamphlet, the capsules contain ginger (*Zingiber officinale*) (root extract) (guaranteed 12.5 mg (5%) gingerols). We had already reported the GRCE phytochemical analysis and toxicity evaluation in our previous paper [21]. The phytochemical analysis revealed that 10-gingerol (525.418 μg/mL), 6-gingerol (443.182 μg/mL), 8-gingerol (360.897 μg/mL), 6-shogaol (369.816 μg/mL), and 1-Dehydro-6-gingerdione (304.115 μg/mL) were the main compounds of GRCE [21]. Injectable diclofenac sodium and saline solution (0.9%) were also purchased from a local pharmacy in Cluj-Napoca.

### 4.2. Animal Grouping and Experimental Design

Fifty-six white male Wistar-Bratislava rats (300–320 g) were randomized into seven groups (8 rats/group), as shown in Table 1. Our previous research [21] explains in detail the experimental groups and acute inflammation model.

### 4.3. Inflammatory Edema Assessment

Paw edema measurements were performed at 1, 3, 5, 7, and 24 h after AI induction, using a digital plethysmometer (Ugo-Basile, Milan, Italy). The volume of fluid displaced in the paw was recorded to measure the paw edema, and the results are expressed as paw volume. The percentage of inhibition of paw volume was determined using the following formula [51]:

% inhibition = (Vi − Vt)/(Vi − Vc) × 100

where Vi = mean of paw volume in AI group, Vc = mean of paw volume in C group, and Vt = mean of paw volume in a group with the following specific treatments: Diclofenac sodium (D)—AI-D group; ginger root capsule extract (GRCE) in a dose of 100 mg/kg b.w.—AI-GRCE100 group; GRCE in a dose of 200 mg/kg b.w.—AI-GRCE200 group; GRCE in a dose of 100 mg/kg b.w. and D—AI-GRCE100-D; and GRCE in a dose of 200 mg/kg b.w. and D—AI-GRCE200-D.

### 4.4. Blood Samples Collection

At 24 h after carrageenan administration, under light anesthesia (ketamine 20 mg/kg b.w., i.p. and xylazine 2 mg/kg b.w., i.p.), the blood samples were collected from the retro-orbital plexuses of each rat. Afterward, the rats were sacrificed by an overdose of anesthetic. Plasma from each rat was obtained by centrifugation at 4 °C for 20 min at 1620× *g*. The obtained samples were transferred in Eppendorf tubes and stored at −80 °C until further analysis.

### 4.5. Tissue Homogenate

Tissue samples were taken from the right paw of rats from all groups immediately after sacrification. Tissue samples were first weighed and homogenized using an automated Witeg Homogenizer (HG-15D, Wertheim, Germany) in 4 volumes of phosphate-buffered saline solution. Afterward, the probes were centrifuged at 1500× *g* for 15 min at 4 °C, and the resulting clear supernatant was transferred in Eppendorf tubes and stored at −80 °C until further analysis.

### 4.6. Biochemical Assays

The plasma and tissue levels of the pro-inflammatory cytokines (TNF-α, IL-6, and IL-1β), β-Nerve Growth Factor (β-NGF) and Prostaglandin-endoperoxide synthase 2 (COX-2) were measured using the enzyme-linked immunosorbent assay (ELISA) technique (Stat Fax 303 Plus Microstrip Reader, Minneapolis, USA). Commercially available kits (rat TNF-α, IL-6, and IL-1β ABTS ELISA Development kits, PeproTech EC, Ltd., London, UK) were used.

### 4.7. Histopathological Examination

The right hind paws were taken and fixed in 10% formalin. They were then embedded in paraffin, sectioned, stained with hematoxylin and eosin, and finally examined under a light microscope by a pathologist who was not aware of the study groups.

### 4.8. Statistical Analysis

The distribution of raw data of the evaluated biomarkers was tested for normality using the Shapiro–Wilk test, and the violation of theoretical normal distribution was considered when the *p*-values were lower than 0.05. The biomarkers were reported as median [Q1 to Q3] (where Q1 is the 25th percentile and Q3 is the 75th percentile) when the raw data proved not to follow a theoretical normal distribution in at least one group. The overall statistical significance was tested using the Kruskal–Wallis test followed by post hoc analysis (multiple comparisons) whenever statistical significance was obtained. Statistical analysis was performed with the Statistica 13 program (v. 13, StatSoft, St. Tulsa, OK, USA). We graphically represented the distribution of biomarkers following the method proposed by Weissgerber et al. [52].

## 5. Conclusions

In monotherapy at 100 mg/kg b.w. and at 200 mg/kg b.w., ginger root capsule extract provided limited anti-inflammatory effects in carrageenan-induced paw edema. The association of ginger root capsule extract with diclofenac sodium potentiates its anti-inflammatory effect. This association also demonstrated a better antiedematogenic effect even from the first hour, with a higher percentage of inhibition for a dose of 200 mg/kg b.w. at 24 h. The association of ginger root capsule extract with a low dose of diclofenac sodium could be an appropriate combination to decrease the dose used to reduce the serum and tissue levels of inflammatory cytokines, edema and histological changes in acute inflammation. However, additional studies are necessary to achieve the clinical utility of this combination.

## Figures and Tables

**Table 1:** Intervention by study groups.

Group.	Abb.	Intervention|Treatment
1. Control group	C	none|saline solution
2. Acute inflammation (AI) model group	AI	acute paw inflammation (API)|saline solution
3. AI treated with Diclofenac sodium (D)	AI-D	API|5 mg/kg b.w. D after API
4. AI treated with Ginger root capsule extract (GRCE) in a dose of 100 mg/kg b.w.	AI-GRCE100	API|100 mg/kg b.w. GRCE and after API
5. AI treated with GRCE in a dose of 200 mg/kg b.w.	AI-GRCE200	API|200 mg/kg b.w. GRCE and after API
6. AI with GRCE in a dose of 100 mg/kg b.w. and D	AI-GRCE100-D	API|100 mg/kg b.w. GRCE and5 mg/kg b.w. D after API
7. AI with GRCE in a dose of 200 mg/kg b.w. and D	AI-GRCE200-D	API|200 mg/kg b.w. GRCE and5 mg/kg b.w. D after API

b.w. = body weight.

## Data Availability

The presented data will not be publicly available until the associated Ph.D. thesis is publicly defended. The raw data can be obtained upon reasonable request when addressed to Ioana Boarescu (e-mail: ioana.chirila.boarescu@elearn.umfcluj.ro).

## Supplementary Materials

The supporting information can be downloaded at https://www.mdpi.com/article/10.3390/ijms25031781/s1.
