# Supplementary material for: Zingiber officinale Root Capsule Extract Synergistically Enhance the Anti-Inflammatory Effects of Diclofenac Sodium in Experimental Acute Inflammation
Source: Int J Mol Sci. 2024 Feb 1;25(3):1781. doi: 10.3390/ijms25031781 (PMC10855350; doi:10.3390/ijms25031781)
Supplement: Supplementary file 1 [file ijms-25-01781-s001.zip › ijms-2843070-supplementary.pdf]

Table S1. Serum values of pro-inflammatory cytokines by group.

Group	TNF-α, pg/mL	IL-6, pg/mL	IL-1β, pg/mL
C	36.1 [24.4 to 47.8]	86.6 [81.5 to 90.5]	117 [105.1 to 120.8]
AI	147.1 [135.6 to 158.1]	229.4 [210.8 to 261.1]	266.2 [244.3 to 334.4]
AI-D	103.9 [99.9 to 107.6]	142.1 [127.5 to 156.8]	178 [164.4 to 202.2]
AI-GRCE100	124.1 [116.5 to 135.1]	208.2 [176 to 222]	216.8 [205 to 288.5]
AI-GRCE200	114.5 [107.8 to 121.7]	181 [169.2 to 184.4]	211.7 [189 to 237.7]
AI-GRCE100-D	81.9 [77 to 88.3]	113 [109.1 to 120.6]	157.7 [151.8 to 162.5]
AI-GRCE200-D	52.9 [47.7 to 57.8]	89.6 [69.1 to 105.2]	133.2 [110.2 to 136.8]
*Stat. (p-value)	50.2 (<0.0001)	48.6 (<0.0001)	44.7 (<0.0001)

Results are expressed as median [Q1 to Q3], where Q is the quartile.. * Kruskal-Wallis test. Abbreviations: C—control; AI—Acute inflammation; D—Diclofenac; GRCE— Ginger root capsule extract.

Table S2. Serum values of β-Nerve Growth Factor (β-NGF) and Prostaglandin-endoperoxide synthase 2 (COX-2) by group.

Group	β-NGF, pg/mL	COX-2, pg/mL
C	130.2 [129.2 to 131.8]	13.8 [8.8 to 18.1]
AI	133.9 [133.5 to 134.8]	124.8 [108.8 to 135.3]
AI-D	132.8 [131.3 to 133.3]	74.8 [65.8 to 78.5]
AI-GRCE100	132.7 [131.6 to 134.6]	112.8 [100.9 to 123.1]
AI-GRCE200	132.0 [131.1 to 133.2]	82.8 [79.3 to 89.3]
AI-GRCE100-D	131.1 [130.5 to 131.8]	38.4 [36.8 to 47.8]
AI-GRCE200-D	130.6 [129.5 to 131.7]	17.8 [15.3 to 23.8]
*Stat. (p-value)	19.6 (0.0033)	49.6 (<0.0001)

Results are expressed as median [Q1 to Q3], where Q is the quartile. * Kruskal-Wallis test. Abbreviations: C—control; AI—Acute inflammation; D—Diclofenac; GRCE— Ginger root capsule extract

Table S3. Tissue values of pro-inflammatory cytokines by group.

Group	TNF-α, pg/mL	IL-6, pg/mL	IL-1β, pg/mL
C	88.5 [82.1 to 94.1]	93.5 [88.9 to 123.1]	204.3 [163.9 to 252.9]
AI	445.7 [378.7 to 570.1]	458.7 [292 to 492.4]	592.6 [453.2 to 661.2]
AI-D	259.7 [188.9 to 313.7]	154.7 [126.6 to 181]	427.2 [323.5 to 452.7]
AI-GRCE100	308.9 [237.8 to 390.6]	227 [162.1 to 291.1]	512.7 [434 to 562.9]
AI-GRCE200	255.3 [220.3 to 280.4]	185.2 [129.7 to 269.8]	427.9 [322.1 to 577.4]
AI-GRCE100-D	185.6 [162.4 to 194.7]	133.1 [118.9 to 149.8]	316.4 [306 to 448]
AI-GRCE200-D	114.9 [110.7 to 126.7]	112.7 [100.3 to 122.8]	234.6 [183.6 to 311.9]
*Stat. (p-value)	43.9 (<0.0001)	33.7 (<0.0001)	29.5 (<0.0001)

Results are expressed as median [Q1 to Q3], where Q is the quartile. * Kruskal-Wallis test. Abbreviations: C—control; AI—Acute inflammation; D—Diclofenac; GRCE— Ginger root capsule extract.

Table S4. Tissue values of β-Nerve Growth Factor (β-NGF) and Prostaglandin-endoperoxide synthase 2 (COX-2) by group.

Group	β-NGF, pg/mL	COX-2, pg/mL
C	147.2 [144 to 176.4]	380.8 [186.4 to 408.8]
AI	480.4 [372 to 585.1]	2055.2 [1606.6 to 2605.5]
AI-D	442 [345.6 to 487.4]	1225.6 [846.6 to 1384.7]
AI-GRCE100	470.7 [354.7 to 503.6]	1813.4 [1285.6 to 2146.2]
AI-GRCE200	444.1 [336.7 to 532.7]	1331.6 [1178.4 to 1559.3]
AI-GRCE100-D	415 [342.5 to 523.7]	909.6 [785.6 to 1032.3]
AI-GRCE200-D	399.7 [268.2 to 527.7]	691.2 [549.5 to 785.3]
*Stat. (p-value)	8.9 (0.1797)	44.6 (<0.0001)

Results are expressed as median [Q1 to Q3], where Q is the quartile. * Kruskal-Wallis test. Abbreviations: C—control; AI—Acute inflammation; D—Diclofenac; GRCE— Ginger root capsule extract
